# Over-expression of the long non-coding RNA HOTTIP inhibits glioma cell growth by BRE

**DOI:** 10.1186/s13046-016-0431-y

**Published:** 2016-10-12

**Authors:** Li-Min Xu, Lei Chen, Feng Li, Run Zhang, Zong-yang Li, Fan-Fan Chen, Xiao-Dan Jiang

**Affiliations:** 1 The National Key Clinic Specialty, The Neurosurgery Institute of Guangdong Province, Guangdong Provincial Key Laboratory on Brain Function Repair and Regeneration, Department of Neurosurgery, Zhujiang Hospital, Southern Medical University, Guangzhou, 510282 China; 2 Department of Neurosurgery, Shenzhen Second People’ s Hospital, The First Affiliated Hospital of Shenzhen University, Shenzhen, 518029 China; 3 Department of Neurosurgery, Guangzhou First People’s Hospital, Guangzhou, 510180 China

**Keywords:** HOTTIP, Glioma, BRE, Apoptosis

## Abstract

**Background:**

Gliomas are the most common type of primary brain tumour in the central nervous system of adults. The long non-coding RNA (lncRNA) HOXA transcript at the distal tip (HOTTIP) is transcribed from the 5′ tip of the HOXA locus. HOTTIP has recently been shown to be dysregulated and play an important role in the progression of several cancers. However, little is known about whether and how HOTTIP regulates glioma development.

**Methods:**

In this study, we assayed the expression of HOTTIP in glioma tissue samples and glioma cell lines using real-time polymerase chain reaction and defined the biological functions of HOTTIP using the CCK-8 assay, flow cytometry, terminal deoxynucleotidyl transferase dUTP nick end labelling (TUNEL assay) and tumour formation assay in a nude mouse model. Finally, we discovered the underlying mechanism using the Apoptosis PCR 384HT Array, Western blot, RNA immunoprecipitation (RIP) and luciferase reporter assay.

**Results:**

HOTTIP was aberrantly down-regulated in glioma tissues and glioma cell lines (U87-MG, U118-MG, U251 and A172), and over-expression of HOTTIP inhibited the growth of glioma cell lines in vitro and in vivo. Furthermore, HOTTIP could directly bind to the brain and reproductive expression (BRE) gene and down-regulate BRE gene expression. In addition, we further verified that over-expression of the BRE gene promoted the growth of glioma cell lines in vitro. Finally, over-expression of HOTTIP significantly suppressed the expression of the cyclin A and CDK2 proteins and increased the expression of the P53 protein. However, we found that the over-expression of BRE significantly increased the expression of the cyclin A and CDK2 proteins and suppressed the expression of the P53 protein. Taken together, these findings suggested that high levels of HOTTIP reduced glioma cell growth. Additionally, the mechanism of HOTTIP-mediated reduction of glioma cell growth may involve the suppression of cyclin A and CDK2 protein expression, which increases P53 protein expression via the down-regulation of BRE.

**Conclusions:**

Our studies demonstrated that over-expression of HOTTIP promotes cell apoptosis and inhibits cell growth in U118-MG and U87-MG human glioma cell lines by down-regulating BRE expression to regulate the expression of P53, CDK2 and Cyclin A proteins. The data described in this study indicate that HOTTIP is an interesting candidate for further functional studies in glioma and demonstrate the potential application of HOTTIP in glioma therapy.

**Electronic supplementary material:**

The online version of this article (doi:10.1186/s13046-016-0431-y) contains supplementary material, which is available to authorized users.

## Background

Gliomas are the most frequent and malignant primary brain tumour in adults [1]. Although glioma surgical cure and adjuvant therapies have made progress over the last 20 years, the prognosis of patients with malignant glioma remains grim. The median survival of patients with glioblastoma multiforme (GBM), the most common grade of malignant glioma, is 10 to 12 months [2]. Advances in suitable therapy to increase the survival rate have been limited because the pathophysiological mechanisms are not known. Therefore, it is essential to reveal the mechanisms underlying glioma development and progression to develop effective therapies.

Long non-coding RNAs (lncRNAs) are defined as transcripts containing more than 200 nucleotides and are typically transcribed by RNA polymerase II [3]. The newly discovered lncRNAs exhibit tissue-specific expression patterns [4]. Strong evidence indicates that lncRNAs play important roles in cancer cell growth, survival and migration/invasion [5–7]. Dysregulation of various lncRNAs has been demonstrated in various cancers, such as prostate cancer, breast cancer, hepatocellular carcinoma, oesophageal squamous cell carcinoma and bladder cancer [8–13]. One such lncRNA, Maternally Expressed Gene 3 (MEG3), demonstrates markedly decreased expression in glioma, gastric cancer and non-small cell lung cancer tissues compared with adjacent normal tissues. Moreover, ectopic expression of MEG3 inhibited cell proliferation and promoted cell apoptosis in various types of cancer cells [13–15].

HOXA distal transcript antisense RNA (HOTTIP) is an antisense non-coding transcript located at the distal end of the HOXA gene cluster. HOTTIP is associated with the PRC2 and WDR5/MLL1 chromatin-modifying complexes and directly binds WDR5 [16]. HOTTIP has recently been found to play important roles in cancer cell growth, survival and migration/invasion. HOTTIP is significantly down-regulated in Hirschsprung (HSCR) disease compared with controls, and knock-down of HOTTIP reduced cell migration and proliferation [17]. Interestingly, HOTTIP is significantly up-regulated in hepatocellular carcinoma (HCC) specimens compared with controls [18]. HOTTIP exhibited tissue-specific expression patterns in HSCR and HCC. However, HOTTIP levels in gliomas tissues and the underlying role and mechanism of HOTTIP in gliomas remain unknown.

The brain and reproductive expression (BRE) gene is expressed in a variety of tissues, including the brain, ovary, testis, heart, kidney, and adrenal glands [19]. BRE is highly expressed in the reproductive and nervous systems, hence its name. BRE is considered to be a homeostatic or housekeeping protein [20] given that the gene is capable of modulating the action of hormones and cytokines in stress response, cell survival, and various pathological conditions, such as cancers [21]. BRE promotes cell survival in lung tumours, hepatocellular carcinoma and oesophageal carcinoma [21–24]. Nevertheless, the function of BRE in relation to the HOTTIP gene in glioma cells has not been investigated.

In this study, we show that HOTTIP expression is down-regulated in glioma tissues and glioma cell lines. HOTTIP may act as a tumour suppressor in glioma cells in vitro and in vivo. Importantly, mechanistic analysis reveals that HOTTIP suppresses cyclin A and CDK2 protein expression and increases P53 protein expression by directly binding and inhibiting the expression of BRE.

## Methods

### Patient samples and cell lines

All of the patients were recruited from the Department of Neurosurgery, Zhujiang Hospital, Southern Medical University between 2014 and 2015. The glioma and normal tissues were immediately stored in liquid nitrogen until total RNA was extracted. All of the patients’ clinical pathology information were obtained from the Institute of Pathology at Zhujiang Hospital (Table 1). The tumour histopathologic diagnoses were graded according to the WHO criteria [25]. The tissues resected from patients of cerebral trauma as normal tissues. All of the patients provided written informed consent to this study, which was approved by the Ethics Committee of the University Hospital of Zhujiang (Number:20150013). There was no selection bias in the glioma sample collection for this study. All of the patients in this study met the following criteria: the glioma diagnosis was appraised by pathological examination, no any anticancer treatment occurred before biopsy collection, and exhaustive clinical-pathologic and further data were available. Human glioma A172, U251, U87-MG and U118-MG cells were purchased from the Institute of Biochemistry and Cell Biology of the Chinese Academy of Sciences (Shanghai, China ATCC). They were maintained in DMEM medium containing 10 % FBS (Gibco, Carlsbad, CA) and cultured at 37 °C with 5 % CO_2_.

**Table 1:** Clinico-pathological factors of 85 patients

Clinical factors	Number of cases	% patients
Sex		
Male	47	55.29 %
Female	38	44.71 %
Age		
<60 years	76	89.41 %
≥60 years	9	10.59 %
WHO grade		
Low grade	37	43.53 %
High grade	48	56.47 %

### Generation of cell lines with stable over-expression of HOTTIP

The pLVX-PGK-Puro and pLVX-Hottip-PGK-Puro vectors containing a puromycin resistance marker were designed and synthesized by Biowit Technologies Co., LTD. To generate stable transfectants, empty vector and the pcDNA-hottip vector were transfected into U87-MG and U118-MG cells, respectively. After 48 h, the cells were transferred into one 24-well plate for the selection of stable cell lines in growth medium containing 6 μg/ml puromycin. The stable cell lines were named U87-PGK, U87-HOTTIP, U118-PGK and U118-HOTTIP.

### Apoptosis PCR 384HT array

Total RNA was isolated with TRIzol reagent (Invitrogen, Carlsbad, CA, USA) according to the manufacturer’s protocol. A 1.5-μg sample of total RNA was reverse transcribed in a final volume of 20 μl using specific primers (HOTTIP) under standard conditions using the PrimeScript RT reagent kit (Thermo Scientific, Shanghai, China). Mix the 2× SuperArray PCR master mix2000 μ, Diluted first strand cDNA synthesis reaction100 μl, ddH_2_O2000 μ, Total 4100 μl Volume in a 5-ml tube. The plate seal of the PCR Array was carefully removed before the addition of the cocktails, and then the 384-Well PCR Array was carefully but tightly sealed with the optical adhesive cover. PCR was conducted at 95 °C for 10 min, followed by 40 cycles of 95 °C for 15 s and 60 °C for 1 min in the fast real-time PCR System. The qPCR results were analyzed and expressed as the relative mRNA expression of the CT (threshold cycle) value, which was then converted to fold changes. We evaluated the expression levels of 370 different key genes involved in apoptosis.

### Transfection of glioma cells

All of the plasmid vectors (pcDNA-BRE and empty vector) for transfection were designed and synthesized by Land Unicome Biological Technology Company. Glioma cells cultured on a six-well plate were transfected with pcDNA-BRE and empty vector using Lipofectamine 2000 (Invitrogen, Guangzhou, China) according to the manufacturer’s instructions. Cells were harvested after 48 h for qRT-PCR and western blot analyses.

### RNA extraction and qRT-PCR analysis

Total RNA was isolated with TRIzol reagent (Invitrogen, Carlsbad, CA, USA) according to the manufacturer’s protocol. Next, 5 μg of total RNA was reverse transcribed in a final volume of 20 μl using specific primers (HOTTIP) or the Oligo(dt) 18 primer (BRE) under standard conditions using the PrimeScript RT reagent kit (Thermo Scientific, Shanghai, China). Assays were performed to detect HOTTIP expression using the PrimeScript RT reagent kit and SYBR Premix Ex Taq (Thermo Scientific, Shanghai, China) according to the manufacturer’s instructions. The relative levels of HOTTIP were determined by qPCR using gene-specific primers. U6 was measured as an internal control because its expression showed minimal variation in different cell lines and cancer specimens. The RT reaction was carried out under the following conditions: 42 °C for 60 min, 70 °C for 5 min, and then holding at 4 °C. After the RT reaction, 1 μl of the complementary DNA was used for subsequent qRT-PCR. The PCR primers for HOTTIP, U6 and BRE were as follows:

- HOTTIP: 5′-AACGATGTGTGTGTGCCTTGAT-3′
- 5′-TGGTCCGACAGGGTGAATT-3′;
- U6: 5′-GCGCGTCGTGTAAAGCGTTC-3′
- 5′-GTGCAGGGTCCGAGGT-3′
- BRE: 5′-GCTGCTGATGTGGAAAGATT-3′
- 5′-AGCTGTCCACTGTTGGTAAAG-3′.

PCR was conducted at 50 °C for 2 min and then 95 °C for 10 min, followed by 40 cycles of 95 °C for 15 s, 60 °C for 34 s, and 72 °C for 30 s using the ABI 7500HT fast real-time PCR System (Applied Biosystems). The qPCR results were analyzed and expressed as the relative mRNA expression of the CT (threshold cycle) value, which was then converted to fold changes.

### Cell proliferation assay

Cell proliferation assays were performed using Cell Counting Kit-8 (Gibco, USA). Cells were plated in 96-well plates (1 × 10^4^ cells/well). CCK-8 (10 μL) was added to each well at various time points (6, 12, 24, 48 and 60 h) and incubated at 37 °C for 2 h. The absorbance of each well at 450 nm was measured using a microplate spectrophotometer (Thermo Multiskan FC; Thermo Fisher, USA). All of the experiments were performed in triplicate.

### Cell cycle and apoptosis analysis

Cells were cultured in six-well plates. After 48 h, the cells were harvested by trypsin and washed twice with phosphate-buffered saline (PBS). Flow cytometric analysis was applied using the Annexin V-FITC/PI Apoptosis Detection Kit (KeyGEN Biotech, Nanjing, China) according to the manufacturer’s instructions. The acquisition and analysis were performed using FACS Calibur Flow Cytometer (Beckman Coulter, Atlanta, GA, USA). All of the experiments were performed in triplicate. Data were expressed as the mean ± SD. Significant differences among the groups were assessed by one-way ANOVA. All of the statistical analyses were carried out using SPSS version 13.0 (SPSS, Chicago, IL, USA). The results were considered to be statistically significant at *P* < 0.05.

### Protein extraction and western blotting

Cells were cultured for 48 h at 37 °C with 5 % CO_2_ and were lysed in RIPA buffer containing protease inhibitor cocktail (Roche Switzerland) using standard procedures. The concentrations of total protein were measured using the BCA Protein Assay Kit (Thermo). Typically, 20 μg of the protein was separated using SDS-PAGE and was transferred to nitrocellulose membranes. The membranes were blocked with 5 % non-fat dry milk for 1 h at room temperature and were incubated with specific antibodies at 4 °C overnight. Sequentially, the secondary antibodies were conjugated to horseradish peroxidase, and the western blot bands were visualized using the Millipore ECL Western Blotting Detection System (Billerica, MA, USA). β-Actin (1:500 Abcam) was used to normalize the quantity of the protein. Immunoblotting was performed using the primary antibodies human anti-BRE (1:1000, Abcam), human anti-P53 (1:1000, Abcam), human anti-CDK2 (1:1000, Abcam) and human anti-Cyclin A (1:1000, Abcam).

### Luciferase reporter assay

We cloned the BRE response element (wide type or mutated), contained in the 3′-untranslated regions (3′-UTR) of HOTTIP, into target sequence of psiCheck2 plasmid, which is downstream of the luciferase reporter gene. Using a luciferase assay kit (Promega, Madison, WI, USA), luciferase activity was measured and target effect was expressed as relative luciferase activity of the reporter vector with target sequence. After incubating for 48 h, the cells were lysed in 1× Passive lysis and assayed with the Dual-Luciferase Reporter Assay System (Promega) to measure the Renilla luciferase activity, with firefly luciferase serving as a transfection control.

### RNA immunoprecipitation

RNA immunoprecipitation (RIP) was performed using the Magna RIP TM RNA-Binding Protein Immunoprecipitation Kit (Millipore, Billerica, MA, USA) and BRE (Abcam) antibody according to the manufacturer’s instructions. Briefly, cells were lysed in RIP lysis buffer, then 100 μl of whole cell extract was incubated with RIP buffer containing A + G magnetic beads conjugated with human BRE antibody, normal IgG (Millipore) as a negative control and Anti-snRNP70 as a positive control (Millipore). Samples were incubated with Proteinase K with shaking to digest the protein and then immunoprecipitated RNA was isolated, The coprecipitated RNAs were detected by reverse transcription PCR. The prime for detecting HOTTIP as follow:

- Sense, AACGATGTGTGTGTGCCTTGAT;
- Antisense, TGGTCCGACAGGGTGAATT.

### Immunohistochemical staining

The mouse tumor tissues were paraffin-embedded and cut into 5-μm-thick slides for immunohistochemical analysis. The slides with tumor sections were performed in 10 mmol/L citrate buffer (pH = 6.0) in a microwave oven for 20 min to expose the antigens. Then, the slides were incubated with primary antibodies (Rabbit BRE antibody, Abcam, 1:100) overnight at 4 °C. The next day, the slides were washed three times in PBS and immunostained with a goat anti-rabbit secondary antibody (Abcam,0.2 ug/ml) for 2 h at room temperature. Finally, slides were stained with diaminobenzidine, and the nucleus was counterstained with hematoxylin. The staining density was evaluated according to the Imagepro plus 6.0.

### Terminal deoxynucleotidyl transferase dUTP nick end labelling

For the detection of apoptosis, cells were stained with the In Situ Apoptosis Detection Kit (Roche, South San Francisco, CA, USA) following the manufacturer’s instructions. Briefly, cells were treated with 4 % paraformaldehyde at room temperature for 30 min and then with 0.3 % H_2_O_2_ at room temperature for 30 min to inactivate endogenous peroxidase, followed by 0.1 % Triton X-100 for 30 min. The cells were next incubated at 37 °C for 1 h with terminal deoxynucleotidyl transferase (TdT) and then with an anti-digoxin combination for 30 min. DAPI (0.1 g/mL) was added to stain the cell nucleus, and the cells were photographed using a fluorescence microscope.

### Tumour formation assay in a nude mouse model

Male athymic BALB/c nude mice aged 4 weeks were bred under SPF conditions and were cared at the NanFang Medical Experimental Animal Care Commission according to their protocols. One million of each of group cells were subcutaneously injected into a single side of the posterior flank of each mouse. After post injection 4 weeks, the mice were killed, and the weights and volumes of each formed tumour were examined. The tumour volumes were counted using the equation V = 0.5 × D × d^2^ (V, volume; D, longitudinal diameter; d, latitudinal diameter) [26]. In this study, the protocol was approved by the Committee on the Ethics of Animal Experiments of Nanfang Medical University (Number:20150024). All surgeries were performed under sodium pentobarbital anaesthesia, and all efforts were made to reduce suffering in the mice [27].

### Statistical analysis

All of the experiments were independently repeated at least three times. Data were expressed as the mean ± SD. Significant differences among the groups were assessed by one-way ANOVA. All of the statistical analyses were carried out using SPSS version 13.0 (SPSS, Chicago, IL, USA). The results were considered to be statistically significant at *P* < 0.05.

## Results

### HOTTIP expression is decreased in glioma samples and cell lines

**Fig. 1:**
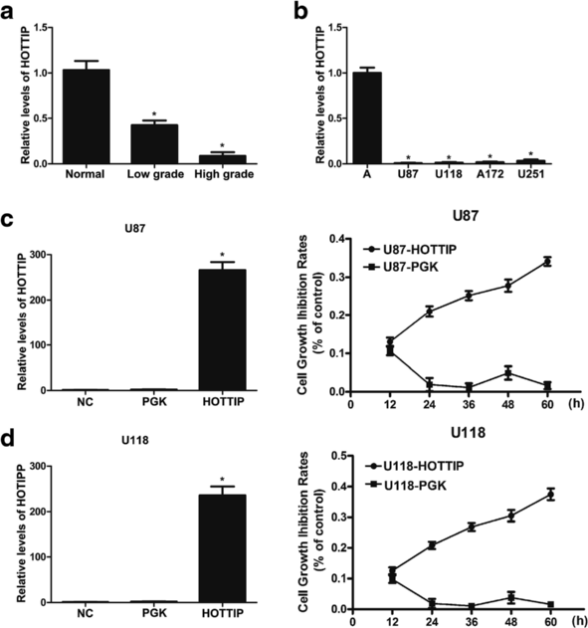
Deregulated expression of the long non-coding RNA HOTTIP in gliomas and glioma cell lines and over-expression of HOTTIP inhibited glioma cell proliferation. **a** HOTTIP expression was assessed by real-time PCR using SYBR Green in human glioma tissues and normal tissues. The 2^−DDCt^ method was used to quantify the relative gene expression levels. **b** HOTTIP expression was assessed by real-time PCR using SYBR Green in four human glioma cell lines (U87-MG, U118-MG, A172 and U251) and astrocyte cells. The 2^−DDCt^ method was used to quantify the relative gene expression levels. **c** Stable over-expression of HOTTIP in U87-MG, and the cck-8 assay was performed to determine proliferation at the indicated time points. **d** Stable over-expression of HOTTIP in U118-MG, and the cck-8 assay was performed to determine proliferation at the indicated time points. The results represent data from at least three independent experiments expressed as the mean ± SD.*P <0.05

To identify the role of HOTTIP in glioma, we analysed the expression of HOTTIP in 85 human glioma tissue samples and 15 human normal brain tissue samples using quantitative real-time RT-PCR. Statistically, average HOTTIP expression levels were decreased in glioma tissue samples; particularly, the expression of HOTTIP was significantly decreased in high-grade glioma tissue samples (Fig. 1a). We also evaluated the expression of HOTTIP in four glioma cell lines (U251, U87, A172, and U118) and immortalized human astrocytes using qRT-PCR. HOTTIP expression in glioma cell lines was significantly decreased compared with that in immortalized human astrocytes (Fig. 1b).

### Over-expression of HOTTIP inhibits cell proliferation and cell cycle progression and promotes apoptosis

**Fig. 2:**
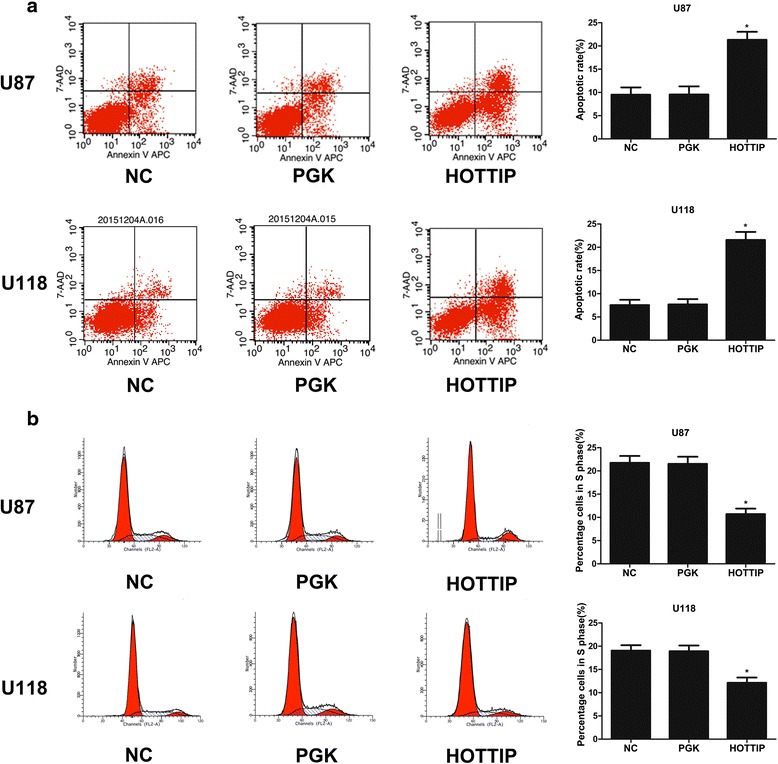
Over-expression of HOTTIP inhibited cell proliferation and promoted apoptosis in the U87-MG and U118-MG glioma cell lines. **a** Stable over-expression of HOTTIP in U87-MG and U118-MG, and the flow cytometry assay was performed to determine cell apoptosis. Over-expression of HOTTIP promoted apoptosis in the U87-MG and U118-MG cell lines. **b** Stable over-expression of HOTTIP in U87-MG and U118-MG, and the flow cytometry assay was performed to assess the cell cycle, Over-expression of HOTTIP reduced percentage of S-phase U87-MG and U118-MG cells. The results represent data from at least three independent experiments expressed as the mean ± SD.*P < 0.05

**Fig. 3:**
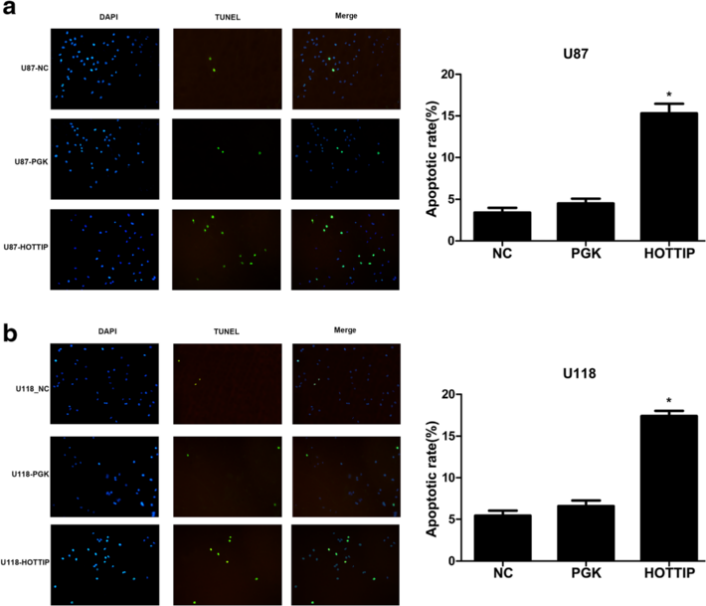
Over-expression of HOTTIP promoted cell apoptosis in the U87-MG and U118-MG glioma cell lines. **a** Stable over-expression of HOTTIP in U87-MG, and the TUNEL assay was performed to assess cell apoptosis. Over-expression of HOTTIP promoted apoptosis in the U87-MG cell line. **b** Stable over-expression of HOTTIP in U118-MG, and the TUNEL assay was performed to assess cell apoptosis. Over-expression of HOTTIP promoted cell apoptosis in the U118-MG cell line. The results represent data from at least three independent experiments expressed as the mean ± SD.*P < 0.05

To investigate the role of HOTTIP in glioma progression, we first established stable over-expression of HOTTIP in the U87-MG and U118-MG cell lines, and CCK-8 assays showed that over-expression of HOTTIP decreased cell proliferation compared with the control group in both cell lines (Fig. 1c, d). To further investigate the role of HOTTIP in glioma progression, we used flow cytometry. Over-expression of HOTTIP led to a decrease in the number of S-phase cells, an increase in the percentage of G0/G1 phase cells, and promoted cell apoptosis (Fig. 2a, b). We confirmed that over-expression of HOTTIP promoted cell apoptosis in U87-MG and U118-MG cells, as assessed by TUNEL staining (Fig. 3a, b). The above findings indicated that over-expression of HOTTIP increases cell apoptosis and inhibits cell proliferation in the two glioma cell lines.

### Association of HOTTIP and BRE

**Fig. 4:**
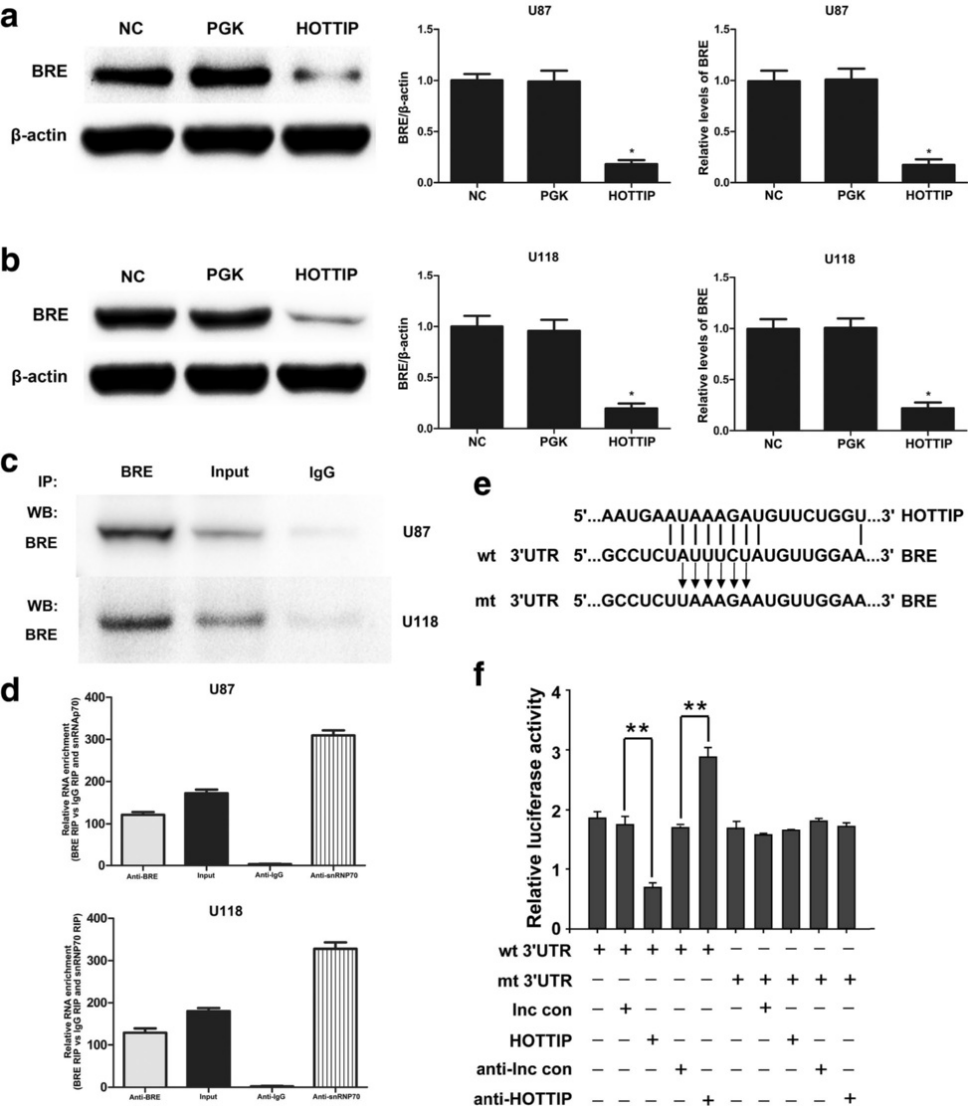
Over-expression of HOTTIP directly decreased BRE expression. **a** and **b** Over-expression of HOTTIP-mediated knockdown of BRE by real-time PCR using SYBR Green and Western blot analysis in the U87-MG and U118-MG glioma cell lines. U6 and β-actin were used as references. The results represent data from at least three independent experiments expressed as the mean ± SD. Significant differences among the groups were assessed by one-way ANOVA. *P < 0.05. **c** Cellular lysates from U87-MG to U118-MG cells were used for RNA immunoprecipitation (RIP) with the BRE antibody. Detection of BRE using IP-Western. **d** RIP was performed using the BRE antibody to immunoprecipitate HOTTIP and a primer to detect HOTTIP. **e** The sequence of BRE response element of wide type (wt) or mutated (mt) and HOTTIP. **f** Luciferase activity of the wild-type binding sites on BRE was significantly suppressed by HOTTIP compared with the lncRNA control. The results represent data from at least three independent experiments expressed as the mean ± SD. (**P < 0.01)

To understand the molecular mechanism by which HOTTIP suppresses glioma cell growth, we detected the expression of 370 different key genes involved in the apoptosis of U87-MG cells over-expressing pcDNA-HOTTIP or empty vector using the Apoptosis PCR 384HT Array. BRE was significantly down-regulated (fold change = 0.14) in U87-MG cells over-expressing HOTTIP compared with empty vector U87-MG cells using the Apoptosis PCR 384HT Array (Table 2; Additional file 1). We generated U87-MG and U118-MG cells that stably over-expressed HOTTIP and found that BRE was significantly decreased in these cells compared with control U87-MG and U118-MG cells using qPCR and Western blotting, indicating that high HOTTIP expression reduces the expression of BRE (Fig. 4a, b). Therefore, we performed RIP with an antibody against BRE using nuclear extracts from U118-MG to U87-MG cells. We observed a significant enrichment of HOTTIP with the BRE antibody compared with the IgG control antibody. To further confirm the association between HOTTIP and BRE, we performed RIP with an antibody against BRE using nuclear extracts from U118-MG to U87-MG cells. We observed a significant enrichment of HOTTIP with the BRE antibody compared with the IgG control antibody (Fig. 4d). Moreover, the HOTTIP levels in immunoprecipitates were determined by qRT-PCR. The HOTTIP levels were enriched in BRE pellets relative to control IgG immunoprecipitates (Fig. 4c). To validate the interactions between BRE and HOTTIP, luciferase reporter assays were performed using the HOTTIP target sequences of wild-type BRE and mutated BRE (Fig. 4e). The luciferase signal was significantly suppressed compared with the lncRNA control, whereas the suppressive effect was not noted when the putative binding site was mutated (Fig. 4f). This, we demonstrated that BRE was post-transcriptionally regulated by HOTTIP in U87-MG glioma cells. These data suggest the association of HOTTIP and BRE, and over-expression of HOTTIP decreased BRE expression in the U87-MGand U118-MG cell lines.

**Table 2:** List of significantly changed (fold change ≥ 1.5) genes involved in apoptosis with over-expression of HOTTIP relative to over-expression of empty vector in U87-MG cells

Genes involved in apoptosis	Fold Difference
ALOX15B	2.57
BCL2A1	1.79
BIRC3	−2.26
BNIP3	1.79
BNIP3L	1.50
BRE	−7.34
CASP5	4.20
CIDEA	3.08
CUL4A	−2.62
DCC	8.17
GSTP1	−4.01
HIPK2	−1.56
IGF1R	−1.56
IL1A	1.97
INHBA	1.83
MAP3K10	−1.56
NLRP3	2.68
NRG2	−1.58
PLAGL1	−2.40
PLAGL2	−3.25
PML	−1.57
POGK	−1.51
POU4F1	3.89
SH3GLB1	−1.54
TBX5	2.41
TIAF1	−2.94
TIMP3	2.54
TNFRSF1B	−1.60
TP63	−3.36
TP73	−1.82
TRADD	1.51

### BRE over-expression promotes cell proliferation and cell cycle progression and inhibits apoptosis

**Fig. 5:**
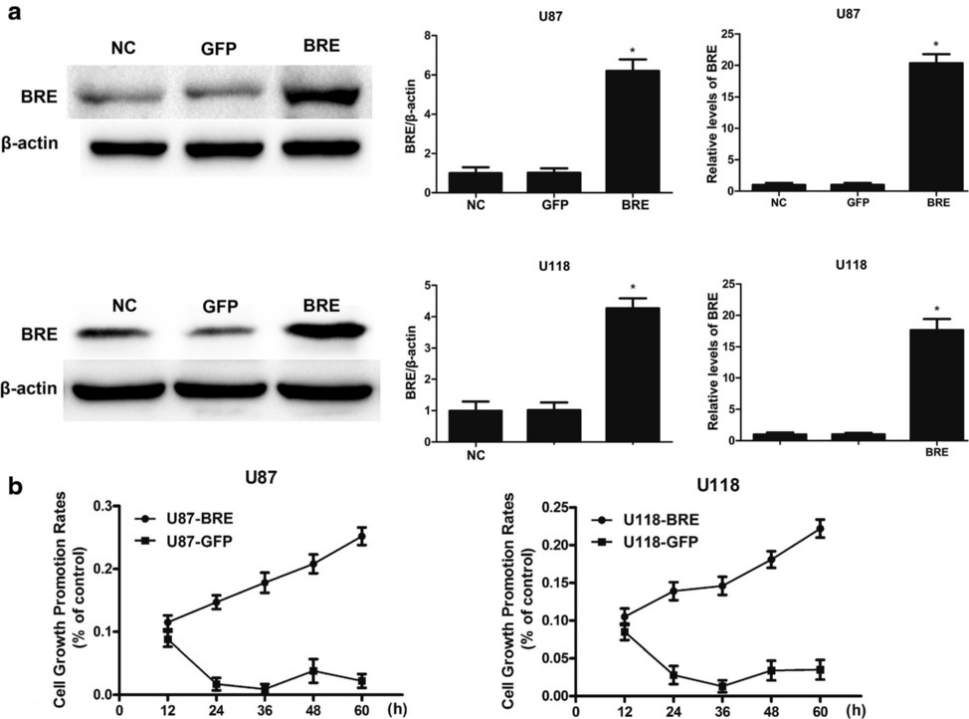
Over-expression of BRE promoted cell proliferation in the U87-MG and U118-MG glioma cell lines. **a** Over-expression of BRE in the U87-MG and U118-MG cell lines. **b** At the indicated time points, cck-8 assays were performed to assess proliferation. The results represent data from at least three independent experiments expressed as the mean ± SD.*P < 0.05

**Fig. 6:**
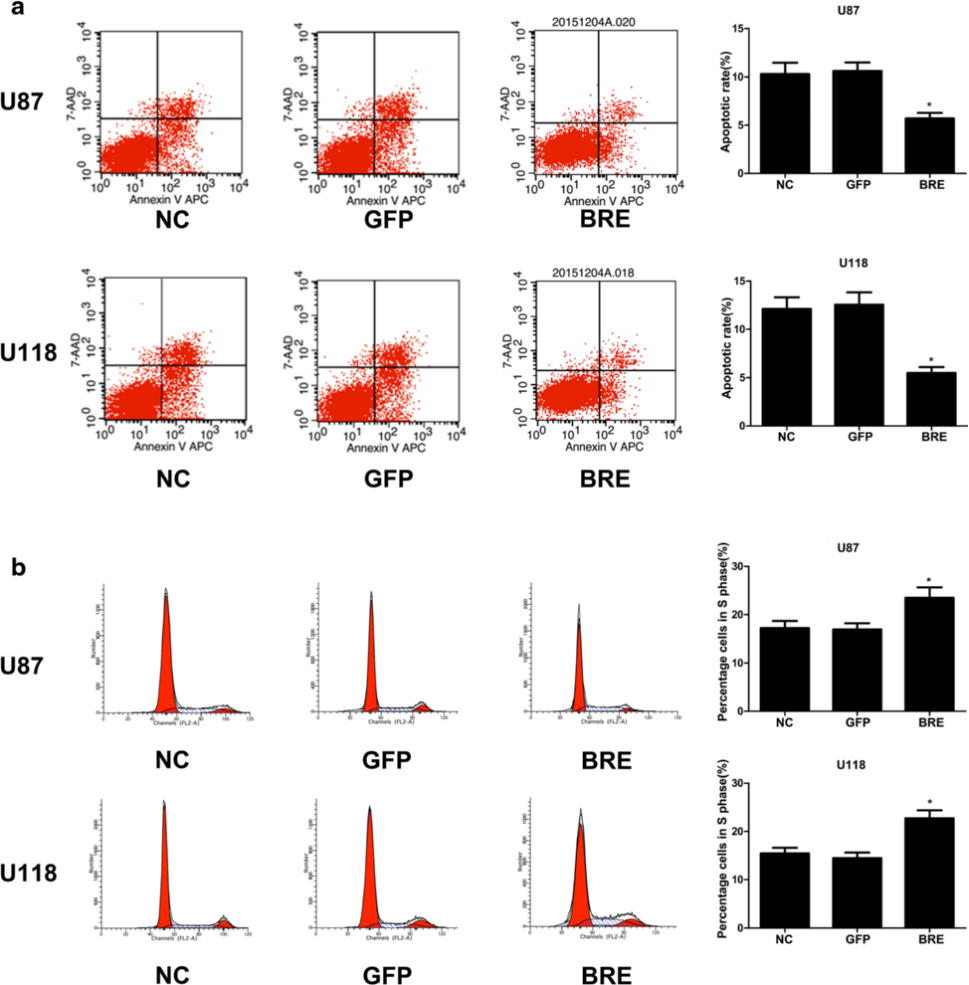
Over-expression of BRE inhibited cell proliferation and promoted cell apoptosis in the U87-MG and U118-MG glioma cell lines. **a** Forty-eight h after transfection of empty vector/pCDNA-BRE in U87-MG and U118-MG, flow cytometry assays were performed to assess cell apoptosis. Over-expression of BRE decreased cell apoptosis in the U87-MG and U118-MG cell lines. **b** Forty-eight h after transfection of empty vector/pCDNA-BRE in U87-MG and U118-MG, flow cytometry assays were performed to assess the cell cycle. Over-expression of BRE increased the percentage of S phase U87-MG and U118-MG cells. The results represent data from at least three independent experiments expressed as the mean ± SD.*P < 0.05

To gain further insight into the function of BRE, BRE was over-expressed in the U87-MG and U118-MG cell lines by transfecting cells with pcDNA-BRE. BRE expression was significantly increased in the U87-MG and U118-MG cell lines after transfection with pcDNA-BRE compared with control cells (Fig. 5a). We revealed that over-expression of BRE significantly promoted cell proliferation in the U87-MG and U118-MG cell lines using CCK-8 assays (Fig. 5b). We observed that over-expression of BRE increased the number of S-phase cells, decreased the percentage of G0/G1-phase cells, and inhibited cell apoptosis, as assessed by flow cytometry (Fig. 6a, b). Our findings demonstrated that the over-expression of BRE promotes cell proliferation, increases the number of S-phase cells, and inhibits cell apoptosis in the U87-MG and U118-MG cell lines.

### Ectopic expression of HOTTIP decreases expression of cyclin A and CDK2 and increases expression of P53 via decreased BRE expression

**Fig. 7:**
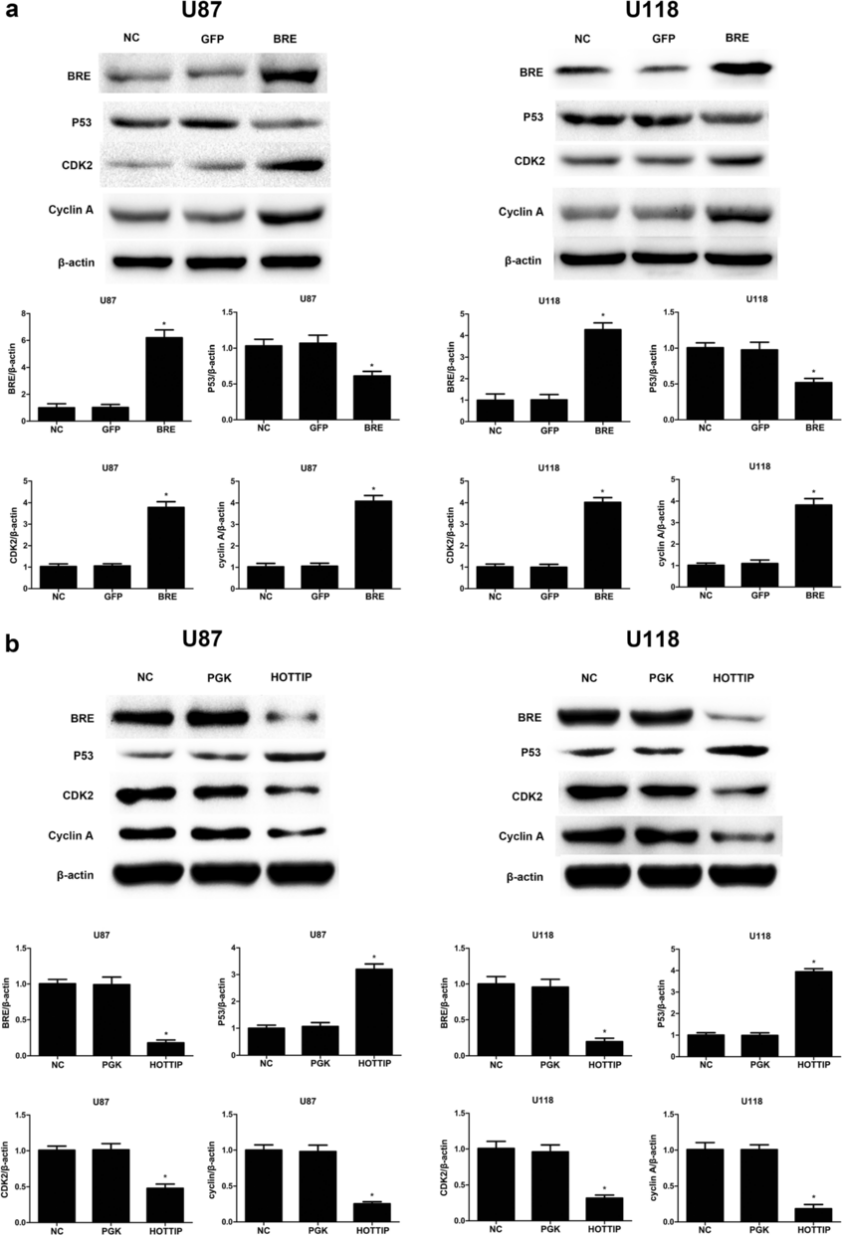
Over-expression of BRE and HOTTIP altered the expression of P53, cyclin A and CDK2 proteins. **a** Over-expression of BRE reduced P53 protein expression and increased cyclin A and CDK2 expression as assessed by Western blot analysis in the U87-MG and U118-MG glioma cell lines. **b** Over-expression of HOTTIP increased P53 protein expression and decreased BRE, cyclin A and CDK2 protein expression as assessed by Western blot analysis in U87-MG and U118-MG glioma cell lines. β-actin was used as a reference. The results represent data from at least three independent experiments expressed as the mean ± SD.*P < 0.05

P53 is an apoptotic signal in various cell types. Cyclins are proteins that act as activators of cyclin-dependent kinases (CDKs) and are required for normal cell cycle transitions. Cyclin A is involved in the transition from G1 to S phase [28]. Hence, to gain further insight into the mechanism of HOTTIP in glioma cell growth and apoptosis, we assessed CDK2, cyclin A and P53 protein expression in each group of cells by Western blotting. The findings indicated that HOTTIP over-expression in U87-MG and U118-MG cells decreased the expression of cyclin A and CDK2 and increased the expression of P53 (Fig. 7b). The over-expression of BRE in U87-MG and U118-MG cells increased cyclin A and CDK2 expression and decreased the expression of P53 (Fig. 7a). All of the above-mentioned findings indicated that the over-expression of HOTTIP might occur by means of down-regulating BRE expression to suppress cyclin A and CDK2 protein expression and increase P53 protein expression.

### Ectopic expression of HOTTIP inhibits the tumourigenesis of U87-MG cells in vivo

**Fig. 8:**
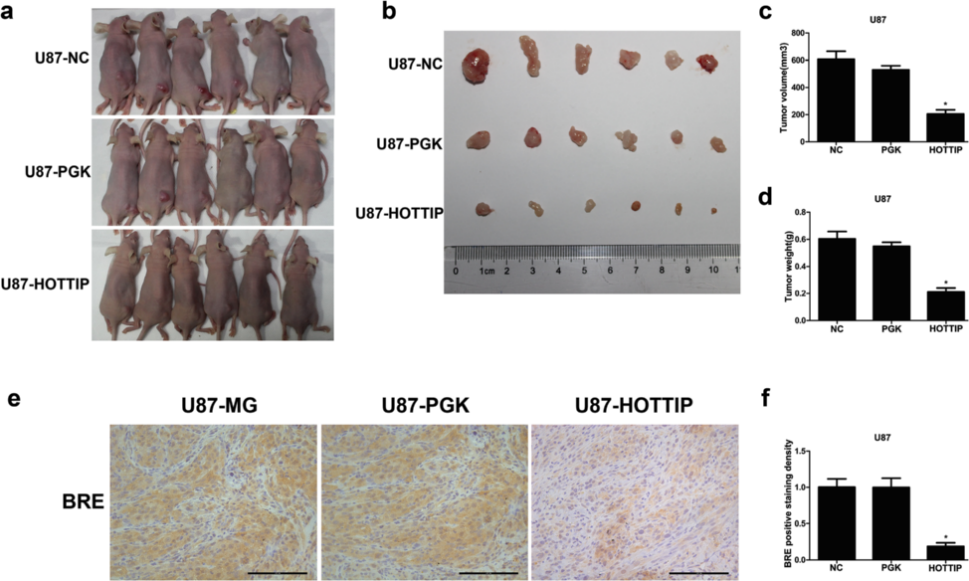
The impact of HOTTIP on tumourigenesis in vivo. **a** and **b** U87-MG cells with stable over-expression of HOTTIP U87-MG or empty vector cells were injected into nude mice (*n* = 6). **c** and **d** Tumour volumes and tumour weights were calculated 4 weeks after injection. **e** and **f** Tumours developed from U87-MG cells stably over-expressing of pCDNA-HOTTIP exhibited reduced BRE protein levels compared with control cells. Error bars indicate the means ± standard errors of the mean. *P < 0.05

To analyse whether HOTTIP affects glioma tumourigenesis in vivo, U87-MG cells and U87-MG cells stably transfected with pcDNA-HOTTIP or empty vector were subcutaneously injected into a single side of the posterior flank of male nude mice. Four weeks after injection, the neoplastic weight and volume in the pcDNA-HOTTIP group were substantially smaller compared with those of the empty vector group and U87-MG group. Additionally, there were no differences between the empty vector group and U87-MG group (Fig. 8a, b, c and d). These results indicated that over-expression of HOTTIP inhibits tumourigenesis in vivo in U87-MG glioma cells. Immunohistochemical staining was used to analyse BRE protein expression in resected tumour tissues. The BRE levels in tumours formed in the pCDNA-HOTTIP group exhibited reduced BEE positivity compared with tumours from the empty vector group and U87-MG group (Fig. 8e, f).

## Discussion

Recently, studies have revealed that the human genome contains more than 20,000 protein-coding genes and that >98 % of the total genome can be transcribed to RNAs that do not produce any proteins. These portions of the genome are named non-coding RNA (ncRNA) genes [29]. lncRNAs are ncRNA transcripts greater than 200 nts in length. There are more than 3000 human lncRNAs, but very few of them have been characterized [30, 31]. Although only a few lncRNAs have been characterized in detail, recent studies have revealed that lncRNAs participate in diverse biological processes through distinct mechanisms [32–34]. However, these molecular mechanisms remain incompletely understood. Thus, more studies should be performed to clarify the biological and molecular mechanisms of lncRNAs in cancer [35].

In this study, we evaluated the expression of HOTTIP in glioma tissues. HOTTIP expression was markedly decreased in glioma tissues compared with normal tissues, and the expression of HOTTIP in glioma cell lines was significantly decreased compared with expression in immortalized human astrocytes. Moreover, ectopic expression of HOTTIP inhibited cell proliferation and decreased the number of S-phase cells in human glioma cell lines, and over-expression of HOTTIP promoted apoptosis in human glioma cell lines. Our results indicate that HOTTIP acts as a tumour suppressor in glioma, which is similar to its role in Hirschsprung (HSCR) disease, but different from its role in hepatocellular carcinoma (HCC). This finding can be attributed to the fact that lncRNAs exhibit tissue-specific expression patterns and lncRNAs can play different roles in different diseases, including cancers [4]. For example, H19, an imprinted gene, has both tumour suppression and oncogenic properties in cancer [36, 37]. The long non-coding RNA TUG1 is up-regulated in hepatocellular carcinoma and promotes cell growth [38], but TUG1 acts as a tumour suppressor in human glioma by promoting cell apoptosis [39]. The long non-coding RNA HOXA11-AS acts as an oncogene in glioma [40], but HOXA11-AS acts as a tumour suppressor gene in epithelial ovarian cancer [41].

Although the majority of lncRNAs have been shown to play important biological roles and are deregulated in many human cancers, the precise molecular mechanisms by which lncRNAs modulate tumour growth remain largely unknown [35]. Ectopic expression of MEG3 inhibited cell proliferation and promoted cell apoptosis via regulation of p53 activation in the U251 and U87-MG human glioma cell lines [15]. However, little is known regarding how HOTTIP regulates glioma development. Brain and Reproductive Organ Expressed (BRE), or BRCC45, is a death receptor-associated antiapoptotic protein [42], and BRE over-expression attenuates cell apoptosis and promotes cell growth in human hepatocellular and oesophageal carcinoma [43, 44]. Are HOTTIP-mediated inhibition of glioma cell proliferation and induction of glioma cell apoptosis related to BRE? BRE was significantly down-regulated (fold change = 0.14) in U87-MG cells over-expressing HOTTIP compared with U87-MG cells with empty vector, as assessed by the Apoptosis PCR 384HT Array. We further demonstrated that over-expression of HOTTIP decreased the expression of BRE mRNA and BRE protein in the U87-MG and U118-MG glioma cell lines, as assessed by qRT-PCR and Western blot, respectively, and we confirmed that HOTTIP directly inhibited BRE expression, as assessed by RIP. Then, we verified that over-expression of BRE promotes cell growth and inhibits apoptosis. Hence, we deduced that over-expression of HOTTIP inhibited glioma cell proliferation and promoted glioma cell apoptosis by down-regulating the expression of BRE.

P53 functions as a crucial tumour suppressor that is involved in mediating tumour suppression. Juanjuan Zhu et al. detected that MEG3 suppressed hepatoma cell growth and promoted cell apoptosis by activating p53-mediated transcriptional activity [45]. Hai Bin Chen et al. demonstrated that the silencing of BRE expression enhanced the expression of p53 protein in human oesophageal carcinoma cells [43]. In this study, we demonstrated that over-expression of HOTTIP increased the expression of P53 and that over-expression of BRE decreased the expression of P53. Thus, we deduced that over-expression of HOTTIP increased the expression of P53 through down-regulation of BRE. Cyclins and CDKs play key roles in cell cycle regulation. In undamaged cells, the cell cycle is controlled by the temporal activation of CDKs (CDK1 and CDK2) in complex with cyclins (cyclin E, A and B) [46]. CDKs and their related pathways regulate the cell cycle by maintaining exit and entry to different phases of the cell cycle [47]. Cyclins are a group of proteins that activate cyclin-dependent kinases and are necessary for normal cell cycle transitions. Cyclin A acts as an activator of two different CDKs (CDK 1 and 2) and impacts both the S and M phases of the cell cycle. Cumulative evidence has demonstrated that the deregulation of cyclins plays a key role in tumour pathogenesis [48]. Simi Santala et al. revealed that high expression of cyclin A is associated with poor prognosis in endometrial endometrioid adenocarcinoma [28]. Evidence indicates that lncRNAs participate in cell cycle regulation. For example, NcRNACCND1 is transcribed from the upstream region of the cyclin D1 gene as a negative regulation factor to regulate cyclin D1 [49]. Gadd7 is an lncRNA that regulates the expression of CDK6 in a posttranscriptional manner [50]. MALAT1 is up-regulated in several human cancers and promotes cancer cell proliferation [51]. MALAT1 depletion suppresses various genes related to cell cycle progression, such as cyclin A2 and Cdc25A, thereby arresting the cell cycle in G1 [52]. Hai Bin Chen et al. demonstrated that silenced expression of BRE inhibits the expression of CDK2 and Cyclin A proteins in human oesophageal carcinoma cells [43]. In this study, we showed that over-expression of HOTTIP can inhibit the expression of CDK2 and cyclin A proteins and that over-expression of BRE can enhance the expression of CDK2 and cyclin A proteins in the U87-MG and U118-MG glioma cell lines. Therefore, we deduced that over-expression of HOTTIP decreased the expression of CDK2 and cyclin A proteins through down-regulation of BRE.

This is the first report to demonstrate the functional significance of HOTTIP expression in glioma, and our findings indicate that HOTTIP functions as a tumour suppressor gene in glioma. Thus, HOTTIP holds great promise as a novel diagnostic and prognostic marker and therapeutic target for glioma.

## Conclusions

Taken together, our studies demonstrated that over-expression of HOTTIP promotes cell apoptosis and inhibits cell growth in U118-MG and U87-MG human glioma cells through down-regulation of BRE expression to regulate the expression of P53, CDK2 and Cyclin A proteins. The data described in this study indicate that HOTTIP is an interesting candidate for further functional studies in glioma and suggest the potential application of HOTTIP in glioma therapy.

## Additional file

Additional file 1: The rezults of apoptosis array. (XLS 63 kb)

## Abbreviations

BRE: Brain and reproductive expression
HOTTIP: HOXA transcript at the distal tip
LncRNA: Long non-coding RNA
MALAT1: Metastasis associated in lung adenocarcinoma transcript 1
MEG3: Maternally Expressed Gene 3
qRT-PCR: quantitative reverse transcription polymerase chain reaction
RIP: RNA immunoprecipitation
Tunnel: Terminal deoxynucleotidyl transferase dUTP nick end labelling

## References

[1] Sathornsumetee S, Rich JN (2006). New treatment strategies for malignant gliomas. Expert Rev Anticancer Ther.

[2] Smith JS (2001). PTEN mutation, EGFR amplification, and outcome in patients with anaplastic astrocytoma and glioblastoma multiforme. J Natl Cancer Inst.

[3] Flynn RA, Chang HY (2014). Long noncoding RNAs in cell-fate programming and reprogramming. Cell Stem Cell.

[4] Guffanti A (2009). A transcriptional sketch of a primary human breast cancer by 454 deep sequencing. BMC Genomics.

[5] Rinn JL (2007). Functional demarcation of active and silent chromatin domains in human HOX loci by noncoding RNAs. Cell.

[6] Gupta RA (2010). Long non-coding RNA HOTAIR reprograms chromatin state to promote cancer metastasis. Nature.

[7] Tsai MC (2010). Long noncoding RNA as modular scaffold of histone modification complexes. Science.

[8] Zhu M (2014). lncRNA H19/miR-675 axis represses prostate cancer metastasis by targeting TGFBI. FEBS J.

[9] Huang J (2014). Long non-coding RNA UCA1 promotes breast tumor growth by suppression of p27 (Kip1). Cell Death Dis.

[10] Hammerle M (2013). Posttranscriptional destabilization of the liver-specific long noncoding RNA HULC by the IGF2 mRNA-binding protein 1 (IGF2BP1). Hepatology.

[11] Chen FJ (2013). Upregulation of the long non-coding RNA HOTAIR promotes esophageal squamous cell carcinoma metastasis and poor prognosis. Mol Carcinog.

[12] Han Y (2013). Hsa-miR-125b suppresses bladder cancer development by down-regulating oncogene SIRT7 and oncogenic long non-coding RNA MALAT1. FEBS Lett.

[13] Lu KH (2013). Long non-coding RNA MEG3 inhibits NSCLC cells proliferation and induces apoptosis by affecting p53 expression. BMC Cancer.

[14] Sun M (2014). Downregulated long noncoding RNA MEG3 is associated with poor prognosis and promotes cell proliferation in gastric cancer. Tumour Biol.

[15] Wang P, Ren Z, Sun P (2012). Overexpression of the long non-coding RNA MEG3 impairs in vitro glioma cell proliferation. J Cell Biochem.

[16] Wang KC (2011). A long noncoding RNA maintains active chromatin to coordinate homeotic gene expression. Nature.

[17] Xie H (2015). Long none coding RNA HOTTIP/HOXA13 act as synergistic role by decreasing cell migration and proliferation in Hirschsprung disease. Biochem Biophys Res Commun.

[18] Quagliata L (2014). Long noncoding RNA HOTTIP/HOXA13 expression is associated with disease progression and predicts outcome in hepatocellular carcinoma patients. Hepatology.

[19] Miao J (2001). Differential expression of a stress-modulating gene, BRE, in the adrenal gland, in adrenal neoplasia, and in abnormal adrenal tissues. J Histochem Cytochem.

[20] Ching AK (2001). Expression of human BRE in multiple isoforms. Biochem Biophys Res Commun.

[21] Tang MK (2006). Comparative proteomic analysis reveals a function of the novel death receptor-associated protein BRE in the regulation of prohibitin and p53 expression and proliferation. Proteomics.

[22] Chan BC (2008). BRE is an antiapoptotic protein in vivo and overexpressed in human hepatocellular carcinoma. Oncogene.

[23] Chan BC (2005). BRE enhances in vivo growth of tumor cells. Biochem Biophys Res Commun.

[24] Chui YL (2010). BRE over-expression promotes growth of hepatocellular carcinoma. Biochem Biophys Res Commun.

[25] Louis DN (2007). The 2007 WHO classification of tumours of the central nervous system. Acta Neuropathol.

[26] Kim K (2013). HOTAIR is a negative prognostic factor and exhibits pro-oncogenic activity in pancreatic cancer. Oncogene.

[27] Kilkenny C (2010). Animal research: reporting in vivo experiments: the ARRIVE guidelines. J Gene Med.

[28] Santala S (2014). High expression of cyclin A is associated with poor prognosis in endometrial endometrioid adenocarcinoma. Tumour Biol.

[29] Consortium EP (2007). Identification and analysis of functional elements in 1 % of the human genome by the ENCODE pilot project. Nature.

[30] Louro R, Smirnova AS, Verjovski-Almeida S (2009). Long intronic noncoding RNA transcription: expression noise or expression choice?. Genomics.

[31] Ponting CP, Oliver PL, Reik W (2009). Evolution and functions of long noncoding RNAs. Cell.

[32] Spitale RC, Tsai MC, Chang HY (2011). RNA templating the epigenome: long noncoding RNAs as molecular scaffolds. Epigenetics.

[33] Wierzbicki AT (2012). The role of long non-coding RNA in transcriptional gene silencing. Curr Opin Plant Biol.

[34] Huang Y (2012). Regulatory long non-coding RNA and its functions. J Physiol Biochem.

[35] Wang F (2015). Upregulated lncRNA-UCA1 contributes to progression of hepatocellular carcinoma through inhibition of miR-216b and activation of FGFR1/ERK signaling pathway. Oncotarget.

[36] Barsyte-Lovejoy D (2006). The c-Myc oncogene directly induces the H19 noncoding RNA by allele-specific binding to potentiate tumorigenesis. Cancer Res.

[37] Colnot S (2004). Colorectal cancers in a new mouse model of familial adenomatous polyposis: influence of genetic and environmental modifiers. Lab Invest.

[38] Huang MD (2015). Long non-coding RNA TUG1 is up-regulated in hepatocellular carcinoma and promotes cell growth and apoptosis by epigenetically silencing of KLF2. Mol Cancer.

[39] Li J (2016). LncRNA TUG1 acts as a tumor suppressor in human glioma by promoting cell apoptosis. Exp Biol Med (Maywood).

[40] Wang Q (2016). A novel cell cycle-associated lncRNA, HOXA11-AS, is transcribed from the 5-prime end of the HOXA transcript and is a biomarker of progression in glioma. Cancer Lett.

[41] Richards EJ (2015). A functional variant in HOXA11-AS, a novel long non-coding RNA, inhibits the oncogenic phenotype of epithelial ovarian cancer. Oncotarget.

[42] Chui YL (2014). Anti-apoptotic protein BRE/BRCC45 attenuates apoptosis through maintaining the expression of caspase inhibitor XIAP in mouse Lewis lung carcinoma D122 cells. Apoptosis.

[43] Chen HB (2008). Comparative proteomic analysis reveals differentially expressed proteins regulated by a potential tumor promoter, BRE, in human esophageal carcinoma cells. Biochem Cell Biol.

[44] Li Q (2004). A death receptor-associated anti-apoptotic protein, BRE, inhibits mitochondrial apoptotic pathway. J Biol Chem.

[45] Zhu J (2015). Long Noncoding RNA MEG3 Interacts with p53 Protein and Regulates Partial p53 Target Genes in Hepatoma Cells. PLoS One.

[46] Sakurikar N (2016). A subset of cancer cell lines is acutely sensitive to the Chk1 inhibitor MK-8776 as monotherapy due to CDK2 activation in S phase. Oncotarget.

[47] Kitagawa M (2013). Cell cycle regulation by long non-coding RNAs. Cell Mol Life Sci.

[48] Stamatakos M (2010). Cell cyclins: triggering elements of cancer or not?. World J Surg Oncol.

[49] Wang X (2008). Induced ncRNAs allosterically modify RNA-binding proteins in cis to inhibit transcription. Nature.

[50] Liu X (2012). Long non-coding RNA gadd7 interacts with TDP-43 and regulates Cdk6 mRNA decay. EMBO J.

[51] Gutschner T, Diederichs S (2012). The hallmarks of cancer: a long non-coding RNA point of view. RNA Biol.

[52] Tripathi V (2013). Long noncoding RNA MALAT1 controls cell cycle progression by regulating the expression of oncogenic transcription factor B-MYB. PLoS Genet.

